# Syncing online: A methodological investigation into movement synchrony, proxemics, and self-other blurring in virtual spaces

**DOI:** 10.1371/journal.pone.0308843

**Published:** 2024-10-22

**Authors:** Manisha Biswas, Marcel Brass

**Affiliations:** 1 Berlin School of Mind and Brain/Department of Psychology, Humboldt-Universität zu Berlin, Berlin, Germany; 2 Department of Experimental Psychology, Ghent University, Ghent, Belgium; 3 Science of Intelligence, Research Cluster of Excellence, Berlin, Germany; Northumbria University, UNITED KINGDOM OF GREAT BRITAIN AND NORTHERN IRELAND

## Abstract

A growing body of literature has found that synchronising movements with a group subsequently increases self-other blurring and social closeness with synchronised partners. However, movement synchrony has not been studied in online settings. Our study has a primarily methodological focus to investigate whether synchronous movement leads to changes in self-other blurring and proxemics in an online, desktop-mediated environment. We conducted two experiments to manipulate synchrony with a group of virtual agents and investigate its impact on self-other blurring and comfort distance judgments. In Experiment 1, we compared synchronous movement to a no-movement condition; in Experiment 2, we introduced an unpredictable movement condition. In both experiments, we found that our manipulation of synchronous movement between participants and a virtual group of agents led to an increase in explicit self-other blurring compared to the no and unpredictable movement conditions; however, we did not find reliable effects on comfort distance judgments.

## Introduction

### Background

Interpersonal synchronous movement is a universal characteristic of human collective behaviour [1] and is often accompanied by a euphoric sense of union [2]. Rituals that incorporate collective movement play a key role in the evolution of human ultrasociality by facilitating the maintenance of large cohesive groups [2–13]. Previous research suggests that interpersonal synchrony expands the contours of the group beyond the confines of familial relationships [14] and physical similarity [15] by utilising behavioural movement patterns as cues of similarity for in-group bond formation [14–21]. In a similar vein, the study of interpersonal space (also known as proxemics), has found that in-group affiliation plays a significant role in how we moderate the space around us [22]. Interpersonal distance [23, 24] and movement synchrony [25, 26] have been previously examined using augmented and virtual reality paradigms. For example, Dotsch & Wigboldus [27] found that Dutch participants maintained more distance and showed higher levels of stress-induced skin conductance when they encountered an avatar with Moroccan facial features as opposed to Caucasian features. Thus, in-group categorisation manifests in terms of interpersonal distance as differing levels of comfort. The closer we feel towards someone the further they are allowed to approach into our personal space (for review [28]). Virtual agents presented via a desktop-computer also impact interpersonal space modulation. For example, Fini et. al. found a social scaling mechanism for extra-personal space in a 3D desktop experiment [29]. Virtual agents capable of movement, as opposed to wooden dummies, were judged as being nearer, indicating a near space extension contingent on the movement potentialities of the other agent. Furthermore, in an immersive virtual reality experiment Tarr et. al found that moving synchronously with virtually presented agents as opposed to asynchronously increases social closeness [26].

### Link between synchrony and proxemics

Shared motor experience leads to remapping of personal space. Following synchronous multisensory stimulation, partners are more readily placed within one’s own peri-personal space [30]. In a study by Jackson et. al, large groups marched either synchronously or asynchronously from one another and in a subsequent dispersal task, groups that marched synchronously tended to stand closer to each other [5]. Furthermore, Tunçgenç & Cohen (2016) found that synchronous movement conditions showed higher bonding, expressed via increased physical proximity in the Island Game [21]. Collectively these studies indicate that the affiliative tendencies that arise out of synchronous movement can be expressed in terms of modulations of interpersonal distance (proxemics). A potential theoretical link between synchrony and interpersonal space modulation is that they both rely on the mechanism of self-other blurring to facilitate affiliation [20, 31–33]. Cooperative and affiliative actions that arise from a ‘*striving to integrate the self into a larger social unit*’ [34] are often expressed in terms of self-other blurring.

Studies that induced synchrony through tapping [7, 35–39], arm-curls [18, 40, 41], bouncing of infants [42–45], limb and arm movement [18, 32], stepping [20], dancing/full body movements [21, 33, 39, 46], singing [9, 35, 47, 48] or swinging [49] found that movement synchronisation is correlated with higher self-reported scores on subjective measures of self-other blurring. Cross et. al. argue that synchrony elicits affiliative tendencies through self-other blurring via recategorization of self and other as common group members, that is, shared synchronous movement facilitates social bonding by re-categorising perceptions of self and co-actor through low level appraisals of similarity [40, 41, 50]. Similarly, research in proxemics has shown that following fair and cooperative interactions in an economic game, peri-personal space boundaries between self and other merged [22]. The existence of this theoretical backing, compounded by the finding that synchronous stimulation [30] and movement [21] leads to modulations of interpersonal space, make a strong case for the investigation of how synchronous movement might impact interpersonal space modulation.

### Online mediated synchrony

The aim of the current study is to investigate whether avatar-mediated synchrony can reliably reproduce affiliative tendencies and to investigate its impact on interpersonal space modulation in an online desktop environment.

In general, social psychological research can be transmitted to the online environments as virtual agents elicit social experiences comparable to real social interactions [23]. Conducting psychological research online offers benefits such as a substantial increase in the number of participants and tightly controlled experiments, ensuring a universal experience for all participants. By leveraging the online nature of our study, we secured a remarkable preregistered sample size (total n = 870 participants), unprecedented in synchrony research.

**Fig 1:**
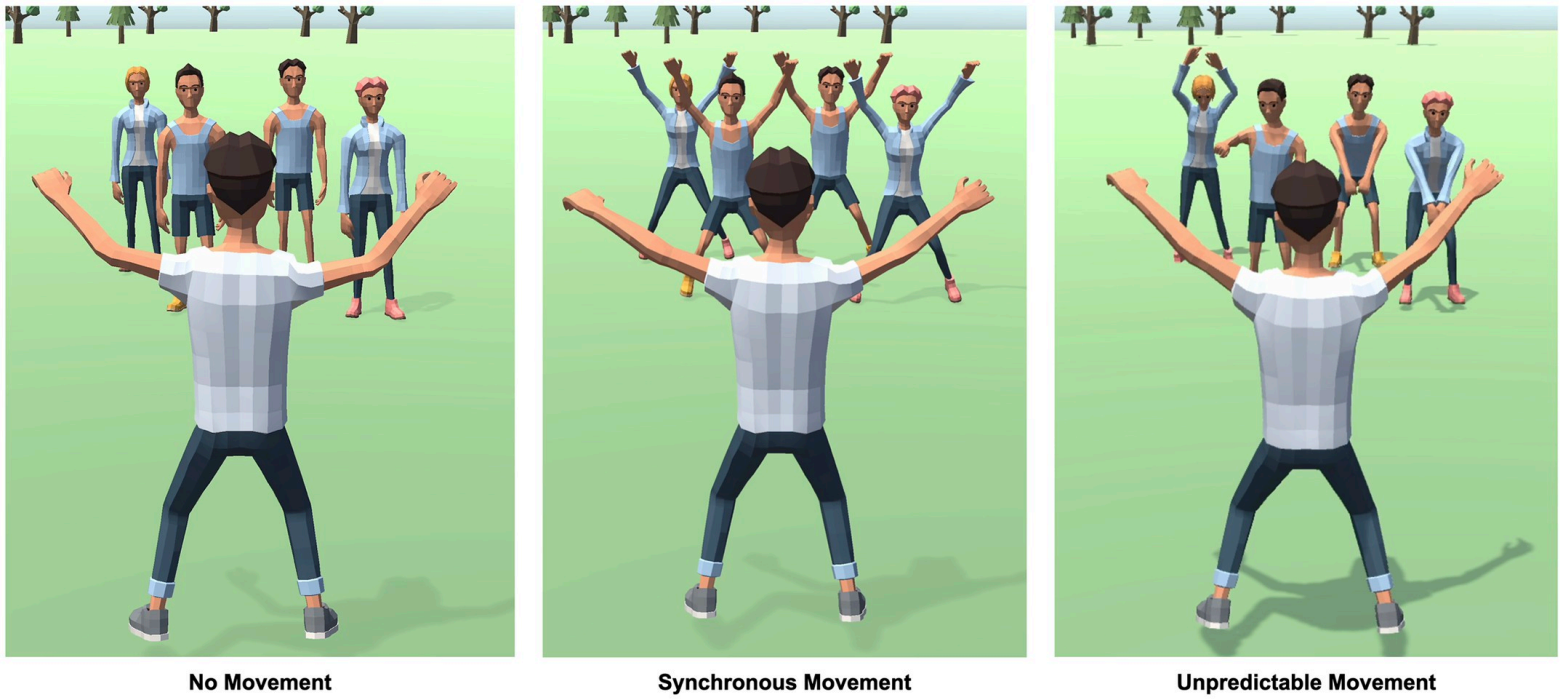
Three experimental conditions (synchronous movement, no-movement, and unpredictable movement) Experiment 1 compared synchronous movement and no movement and Experiment 2 compared all three conditions.

Most studies on synchrony have been conducted in face-to-face and desktop settings wherein participants move their bodies to achieve interpersonal synchrony with virtually presented agents (see for example, [51]). In our study we employ an avatar mediated experience of synchrony, see Fig 1. It is important to consider that there may be psychological differences between a mediated and an unmediated experience of synchronous movement. Unmediated experiences involve participants performing identical movements to the group’s with their own bodies. In contrast, mediated experiences involve no real correspondence of movements between participants and the interacting group presented online. The interpersonal synchrony that emerges only exists between a controlled avatar and a group of agents. We designed our experiment to investigate whether synchrony induced affiliation can occur in mediated settings and to infer the extent of the effects, specifically, whether synchronous movement can produce both explicit self-other blurring and implicit interpersonal space modulation.

### Current study

Our experiment consists of 2 dependent measures; an explicit measure of self-other blurring (IOS Scale) and an implicit measure of proxemics, calculated by averaging the responses on the stop-distance task. It should be also noted that while IOS scale is a widely used indicator of self-other blurring, there are mixed findings regarding its relationship with synchrony. Some studies have found that it did not mediate the relationship between synchrony and in group bonding [13], whereas others did [52–54].

We instructed participants to synchronise the movement of their avatar to a 60-bpm metronome beat by pressing a button. They were not directly instructed to synchronise their movement with the group of virtual agents and the synchrony that arose was incidental. In previous studies it has been shown that synchrony can produce affiliative tendencies even when synchrony is incidental to the main task [36], even though participants realised that this resulting synchrony was only due to external circumstances, they reported liking partners who had been in sync with them more than asynchronous partners. Some studies, however, found no effects or more complex effects relating to incidental synchrony (see [54, 55]).

**Fig 2:**
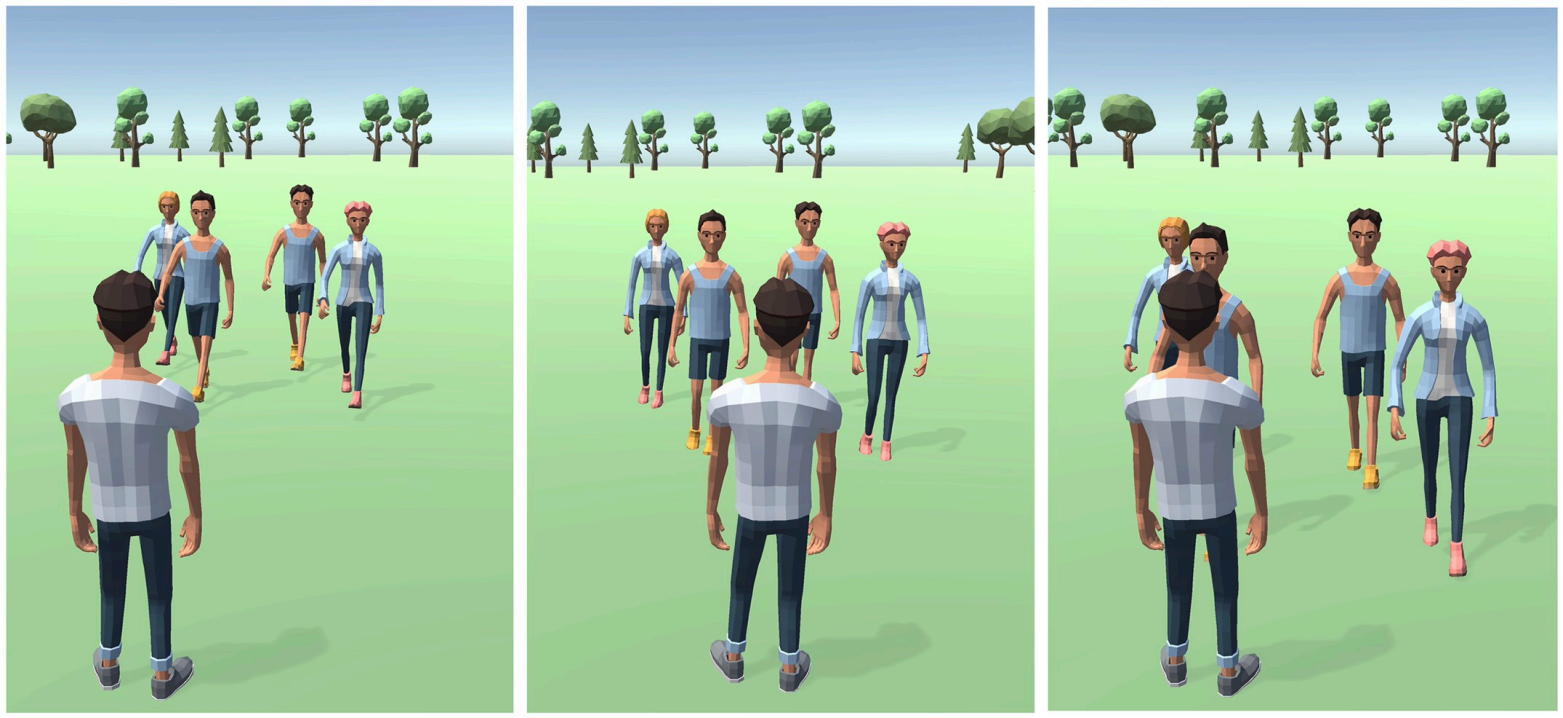
Comfort distance task where participants indicated discomfort as agents approached their avatar. Distances at which participants stopped the agents were recorded to measure comfort distance judgements.

To investigate proxemics, we used the stop distance paradigm [56] sometimes also referred to as the “comfort distance under passive approach” task. In this task a group of agents approaches the participant’s avatar and the participant is instructed to press a button to indicate at which point the diminishing distance between their avatar and the group makes them feel uncomfortable, see Fig 2. In the synchrony condition, we expected to see a comfort distance extension, as opposed to the condition wherein the agent group either showed no movement or moved in an unpredictable manner. That is, our hypothesis was that following shared synchronous movement, the participant would allow the agent group to approach their avatar at a closer distance before reporting discomfort. In the second experiment, to rule out the possibility that the effect was simply due to the presence of movement and not shared synchronous movement we added an unpredictable movement condition, wherein the four characters in the agent group move independently of each other and the participants’ avatar, that is, they performed random actions at random times, see last panel of Fig 1. This condition also allows us to manipulate the predictability of action identity, an essential component of synchronous movement [57]. Even though both movement identity and temporal phasic relations contribute to synchrony, most synchrony paradigms exclusively manipulate the latter of these components by changing the timing of movement in agents to create an asynchronous rhythm. By introducing an unpredictable movement condition, we further extend the current understanding by manipulating both movement identity and time predictability.

## Experiment 1

### Methods

Details of the experiment were pre-registered [https://osf.io/5ytmq].

#### Participants

We collected the data of 457 participants (198 female, 5 non-binary, *M*_*age*_ = 28.7, *SD*_*ag*e_ = 9) from Prolific. 97 participants were excluded based on our pre-registered exclusion criteria (69 for > = 10 comfort distance judgements out of range, 21 for missing > = 3 catch trials and 7 for meeting both criteria). Participants that did not meet the exclusion criteria were replaced to ensure that 360 participants remained after the exclusion criteria were applied (156 female, 5 non-binary, *M*_*age*_ = 28.7, *SD*_*ag*e_ = 9). In accordance with the expected directional effects, we based our sample size on a one tailed dependent t-test power analysis where we estimated a small effect size (d = 0.3) for our implicit dependent measure. All participants were naïve to the aims of the experiment and compensated at a rate of 6 £/hr for their time. Participants provided informed consent before they began the experiment. The experiment was approved by the ethical review board at the Institute of Psychology, Humboldt University of Berlin.

#### Design and procedure

The experiment was programmed on Unity and administered hosted online using JATOS (Just Another Tool for Online Studies). It comprised 2 groups of participants: the experimental group exposed to the synchronous movement condition and the control group exposed to the no movement condition. Each experimental block consisted of 2 parts, Synchrony manipulation and Stop Distance Task. Participants began with a practice block wherein they received feedback (1) if they did not synchronise their button presses with the audio cue and (2) if they did not follow the instructions during the stop distance trials, that is, if they reported discomfort as soon as the trial began or if they did not report until the trial ended (when agent group was face-to-face with their avatar). The practice block was followed by 5 experimental blocks, thus there were a total of 25 stop distance judgments provided by each participant. Following this, the participants answered the IOS (Inclusion-of-other-in-self) scale [31],a self-reported indicator of perceived overlap between self and other. Then participants were redirected to prolific and compensated for their time. The experiment took approximately 15 minutes to complete.

#### Synchrony manipulation

Each block began with the synchrony manipulation, participants were presented with an avatar that they could control by pressing the space bar and they were instructed to press the spacebar when they heard an audio cue. The audio cue was presented 13 times per block at a rhythm of 60 bpm. When participants pressed the spacebar, their avatar moved in the jumping-jacks motion, see Fig 1. The jumping jacks movement animation took approximately 900 ms to complete. A group of 4 agents (2 male, 2 female) faced the participant’s avatar and depending on the condition assigned to the participant, the agents either moved synchronously with their avatar or were in idle pose while their avatar moved. When participants heard the first three beeps and subsequently pressed the button, their avatar moved alone to facilitate embodiment, followed by 10 beeps during which the agent group either moved synchronously with the avatar or did not move, see Fig 1.

#### Stop distance task

In the stop-distance task, the same group of agents that had been previously presented began walking toward the participant’s avatar. Participants were instructed to press the spacebar to stop the approaching group when the diminishing interpersonal distance made them feel uncomfortable. Once the participants reported discomfort, the agent group stopped and then disappeared. In the next trial, they reappeared in their original position and once again began approaching their avatar. Each Stop Distance trial could last up to approximately 5 seconds if the participant did not respond, once the participant responded the trial ended and the next one began. This was repeated 5 times per block. The camera position was jittered for each repetition to prevent participants from explicit strategies, see Fig 2.

#### Statistical analysis

We coded the stop distance task such that the distance between the agent group and the avatar ranged between 0–100. A judgement at 0 was indicative of participant’s waiting too long to report discomfort and a judgement larger than 90 was indicative of the participant reporting discomfort as soon as the trial began. We chose 90 as the threshold because a response earlier than 90 meant that the participant responded before the group had moved from their original position. Thus, as per our pre-registration, judgments that were larger than 90 and at 0 were removed. Valid stop distance trials were used to calculate an average comfort distance per participant. Participant’s response on the Self-Other blurring scale served as their IOS score. Analyses were conducted on Python, R (version 4.2) and Jamovi (version 0.16.2).

### Results

#### Self-other blurring

**Fig 3:**
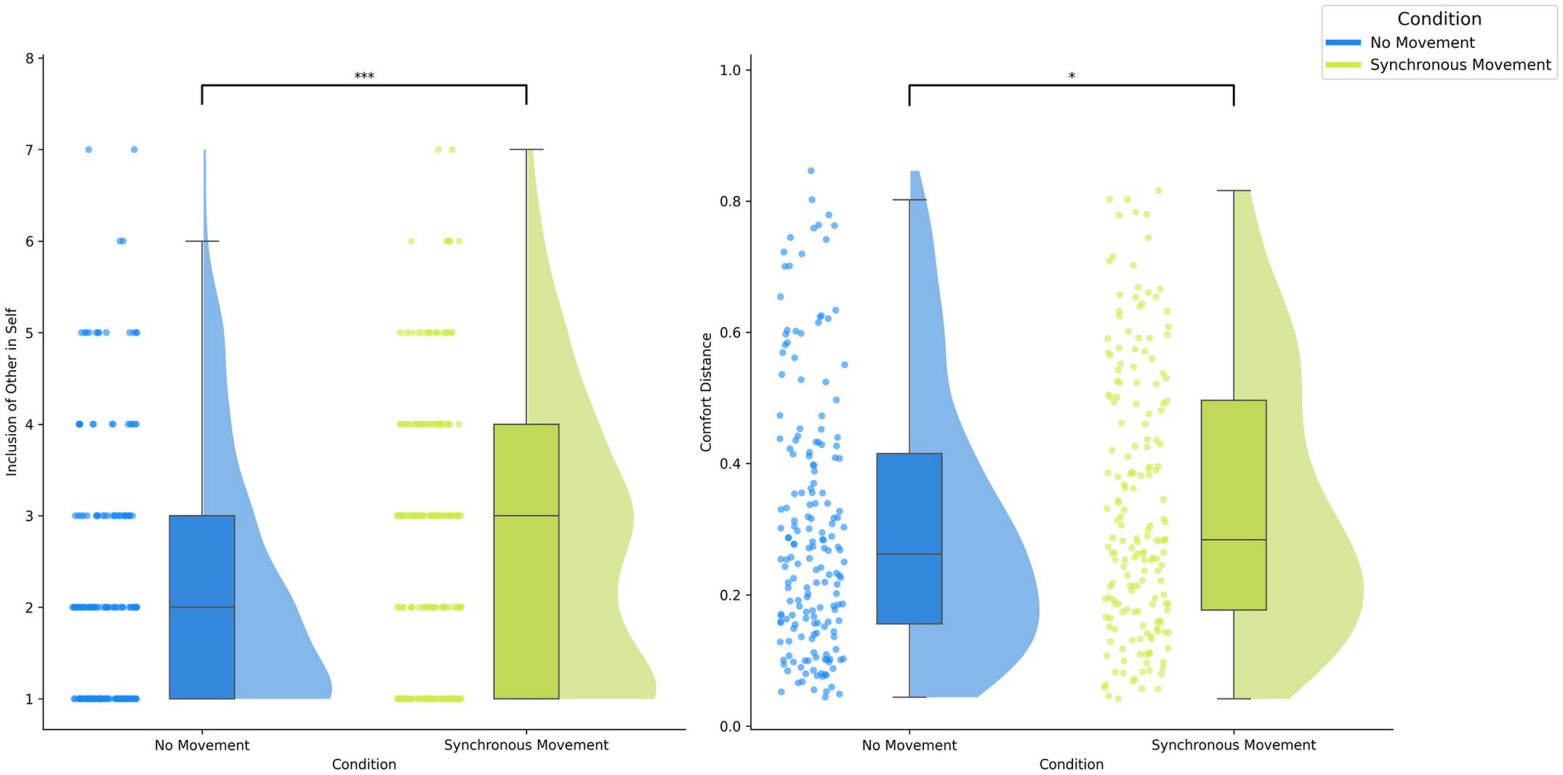
Results from Experiment 1 showing Inclusion of Other in Self (IOS) scores (left) and mean comfort distances (right) for synchronous movement and no-movement conditions. Error bars represent standard errors.

The IOS or Inclusion of Other in Self scale is a self-reported indicator of perceived overlap between self and other (in this case, a group of agents). We compared IOS Scale scores across the Synchronous Movement and No Movement conditions. The IOS scores did not follow a normal distribution as indicated by the Shapiro-Wilk test (Sync: W = 0.883, p < .001; No Movement: W = 0.806, p < .001) and variances were not equal according to Levene’s test (p < .05). Results showed that IOS scores were significantly higher in the Synchronous Movement condition (Sync: *M* = 2.644, *Mdn* = 3, *SD* = 1.519, Range = 1–7) as compared (U = 19,122, p < .001, Z = 2.96) to the No Movement condition (No Movement: *M* = 2.178, *Mdn* = 2, *SD* = 1.383, Range = 1–7) in a directional Mann-Whitney U test (Synchronous Movement > No Movement), see Fig 3.

#### Comfort distance

Mean Comfort Distance refers to the average distance at which the participant stopped the approaching group. Mean Comfort Distances were not normally distributed according to the Shapiro-Wilk test (Sync W = 0.943, p < .001; No Movement W = 0.915, p < .001). The significant effect we found was contrary to that which we hypothesised, i.e., (Synchronous Movement < No Movement). In a Mann-Whitney U test (Synchronous Movement > No Movement), Mean Comfort Distance following the Synchronous Movement (*M* = 0.339, *Mdn* = 0.284, *SD* = 0.200, Range = 0.041–0.816) were significantly greater (U = 17,983, p < 0.05, Z = 1.805) than those following No Movement (*M* = 0.302, *Mdn* = 0.262, *SD* = 0.195, Range = 0.043–0.846), see Fig 3.

### Exploratory analyses

#### Correlating explicit and implicit measures

To establish whether there was a relationship between self-other blurring and comfort distance judgments, we correlated IOS and CD variables across participants. As the data was not normally distributed, we report Spearman’s rank order correlation. To correct for multiple comparisons, we also report corrected p values using the Holm–Bonferroni method.

We found no correlation between them when data was collated across conditions [r = -0.060, 95% CI [0.043, -0.163], p = 0.25]. As pre-registered, we also sub-set the data based on condition. Synchronous Movement: We found no significant correlation between IOS and CD variables when only the synchronous movement condition was analysed [r = 0.066, 95% CI [0.211, -0.081], p = 0.376]. No Movement: We found a significant correlation between IOS and CD variables when only the no movement condition was analysed, [r = -0.226, 95% CI [-0.083, -0.361], p = 0.002, corrected p = 0.006], see Fig 5.

#### Post-task variables: Embodiment, strategy use and purpose check

We pre-registered the intention to check if any of the post-task questions might explain some of the variance in the data. Specifically, we were interested in Embodiment, Strategy Use and Purpose Check. None of these factors had any significant impact on our variables of interest, for a detailed report please consult Section 1 of the supplementary materials.

## Experiment 2

Results from the Experiment 1 indicate that we were able to reproduce self-other blurring in the online context, contrasting our hypothesis we found that comfort distance judgements were greater for following synchronous movement as opposed to no movement. We conducted Experiment 2 to replicate our unexpected finding regarding the relationship between synchronous movement and proxemic judgements and to investigate the relationship between IOS Scores and Comfort distance judgements. Furthermore, the effects found in Experiment 1 might be merely as a result of the perception of movement in the agent group and not synchronous movement per se. We addressed this concern by adding a new condition wherein the agent group moved in an unpredictable manner, that is, they moved at random times and performed a random action from a set of predetermined actions, see last panel of Fig 1.This experiment had one between-subjects factor: Agent Movement which varied at 3 levels, Synchronous movement, No movement and Unpredictable movement. The procedure was the same as Experiment 1. We asked participants to press the spacebar as soon as they heard the audio cue and report discomfort in the stop distance task.

### Methods

Experiment 2 was pre-registered [https://osf.io/n7ruq]. The procedure was the same as Experiment 1.

#### Participants

574 subjects were recruited through prolific. We excluded 64 participants based on pre-registered exclusion criteria (24 for missing > = 3 catch trials, 36 for > 10 comfort distance judgements out of bounds and 4 for meeting both criteria). Excluded subjects were replaced to ensure that 510 subjects remained (245 female, 3 non-binary, 2 no answer; *M*_*age*_ = 37.94, *SD*_*age*_ = 11.20). 170 participants per condition, in accordance with the expected directional effects we based our sample size on a one tailed dependent t-test power analysis of our data from Experiment 1 at a significance level of 0. 025 to correct for multiple comparisons, as we plan to conduct two independent t-tests on the data.

#### Design and procedure

The experiment consisted of three groups of participants: the synchronous movement group, the no movement group, and the unpredictable movement group. As before, each experimental block consisted of two parts: Synchrony manipulation and Stop Distance Task.

#### Synchrony manipulation

Synchrony and No movement condition were identical to Experiment 1. We added a new condition, Unpredictable movement. In this condition, the participant heard the audio cue and when they pressed the spacebar, their avatar moved. The 4 agents facing their avatar moved randomly during the action task, that is each of the agents acted at random times within the block and when they moved, they produced a variety of random motions, see Fig 1. Thus, each of the agents moved independently of each other and independently of the movement produced by the participant’s avatar.

#### Statistical analysis

As we did not have a hypothesis relating to the relationship between No movement and Unpredictable movement condition, we opted to conduct two t-tests. One that compared the No movement and the Synchronous movement conditions, which would help us reproduce our findings from Experiment 1. The other between the Synchronous movement and Unpredictable movement condition which would help us discern whether the effect was due to the mere perception of movement in the agent group. Since we are conducting two t-tests, we will set our significance level at 0.025 to correct for multiple comparisons.

### Results

#### Self-other blurring

**Fig 4:**
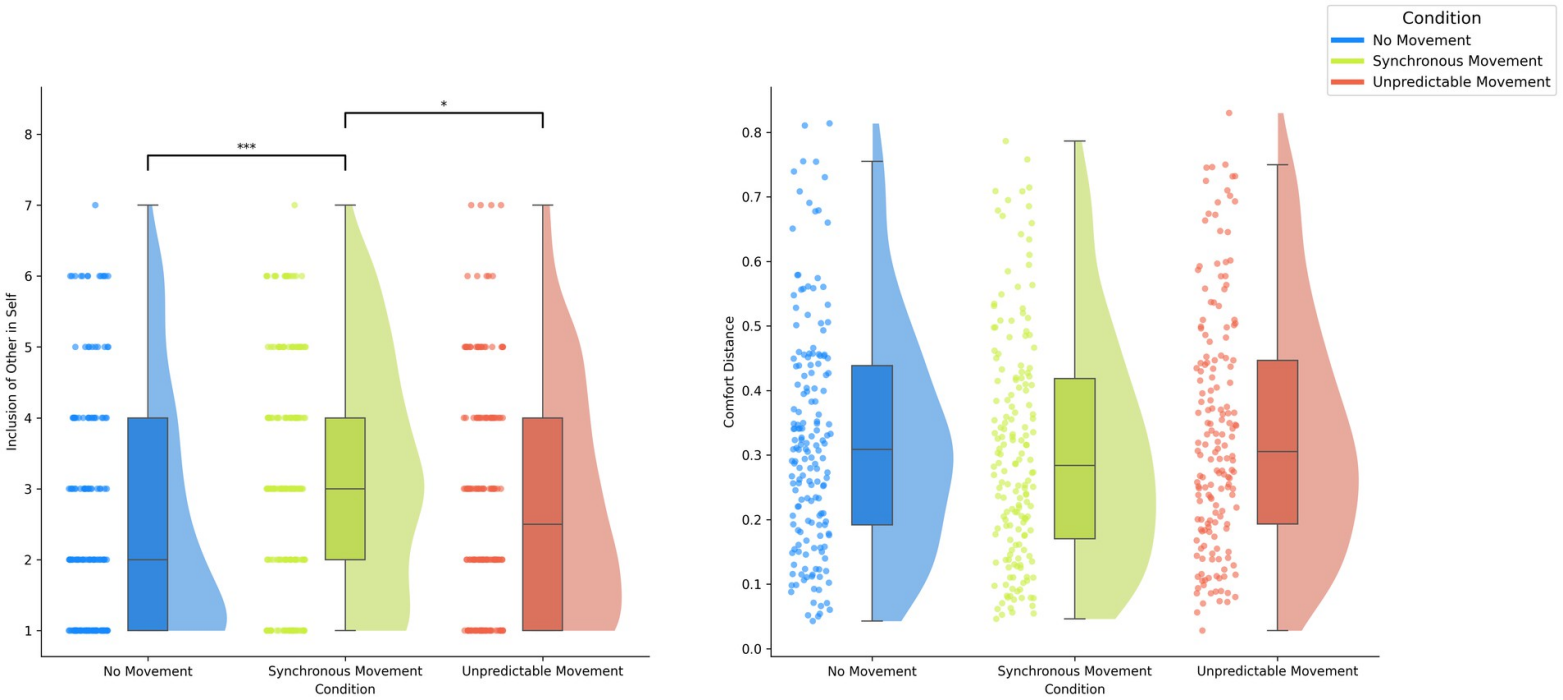
Experiment 2 Results self-other blurring (IOS scores) (left) and mean comfort distance judgements (right) across synchronous movement, no-movement, and unpredictable movement conditions. Error bars represent standard errors.

*Synchrony and no movement*. IOS scores were not normally distributed according to the Shapiro-Wilk test (Sync W = 0.920, p < .001; NoMove W = 0.832, p < .001*)*. Results showed that IOS scores were significantly higher in the Synchronous Movement condition (Sync: *M* = 3.18, *Mdn* = 3, *SD* = 1.61, Range = 1–7) as compared (U = 18,059.5, p < .001, Z = 3.98) to the No Movement condition (No Movement: *M* = 2.5, *Mdn* = 2, *SD* = 1.64, Range = 1–7) in a directional Mann-Whitney U test (Synchronous Movement > No Movement), see Fig 4.

*Synchrony and unpredictable movement*. IOS scores were not normally distributed according to the Shapiro-Wilk test (Sync W = 0.920, p < .001; Unpredictable W = 0.895, p < .001). Results showed that IOS scores were significantly higher in the Synchronous Movement condition (Sync: *M* = 3.18, *Mdn* = 3, *SD* = 1.61, Range = 1–7) as compared (U = 16367.5, p < .05, Z = 2.12) to the Unpredictable Movement condition (Unpredictable: *M* = 2.82, *Mdn* = 2.5, *SD* = 1.60, Range = 1–7) in a directional Mann-Whitney U test (Synchronous Movement > Unpredictable Movement), see Fig 4.

#### Comfort distance

*Synchrony and no movement*. Mean Comfort Distance (CD) scores were not normally distributed according to the Shapiro-Wilk test (Sync W = 0.952, p < .001; No Movement W = 0.958, p < .001). Results showed that Mean Comfort Distances were not significantly lower in the Synchronous Movement condition (Sync: *M* = 0.31, *Mdn* = 0.28, *SD* = 0.18, Range = 0.046–0.786) as compared (U = 13562.0, p = 0.164, Z = -0.98) to No Movement condition (No Movement: *M* = 0.32, *Mdn* = 0.31, *SD* = 0.17, Range = 0.042–0.813) in a directional Mann-Whitney U test (Synchronous Movement < No Movement), see Fig 4.

*Synchrony and unpredictable movement*. Mean Comfort Distance (CD) scores were not normally distributed according to the Shapiro-Wilk test (Sync W = 0.952, p < .001; Unpredictable Movement W = 0.955, p < .001). Results showed that Mean Comfort Distances were not significantly lower in the Synchronous Movement condition (Sync: *M* = 0.31, *Mdn* = 0.28, *SD* = 0.18, Range = 0.046–0.786) as compared (U = 13,132.0, p = 0.073, Z = -1.45) to the Unpredictable Movement condition (Unpredictable: *M* = 0.34, *Mdn* = 0.31, *SD* = 0.18, Range = 0.027–0.829) in a directional Mann-Whitney U test (Synchronous Movement < Unpredictable Movement), see Fig 4.

### Exploratory analyses

#### Co-relating explicit and implicit measures

**Fig 5:**
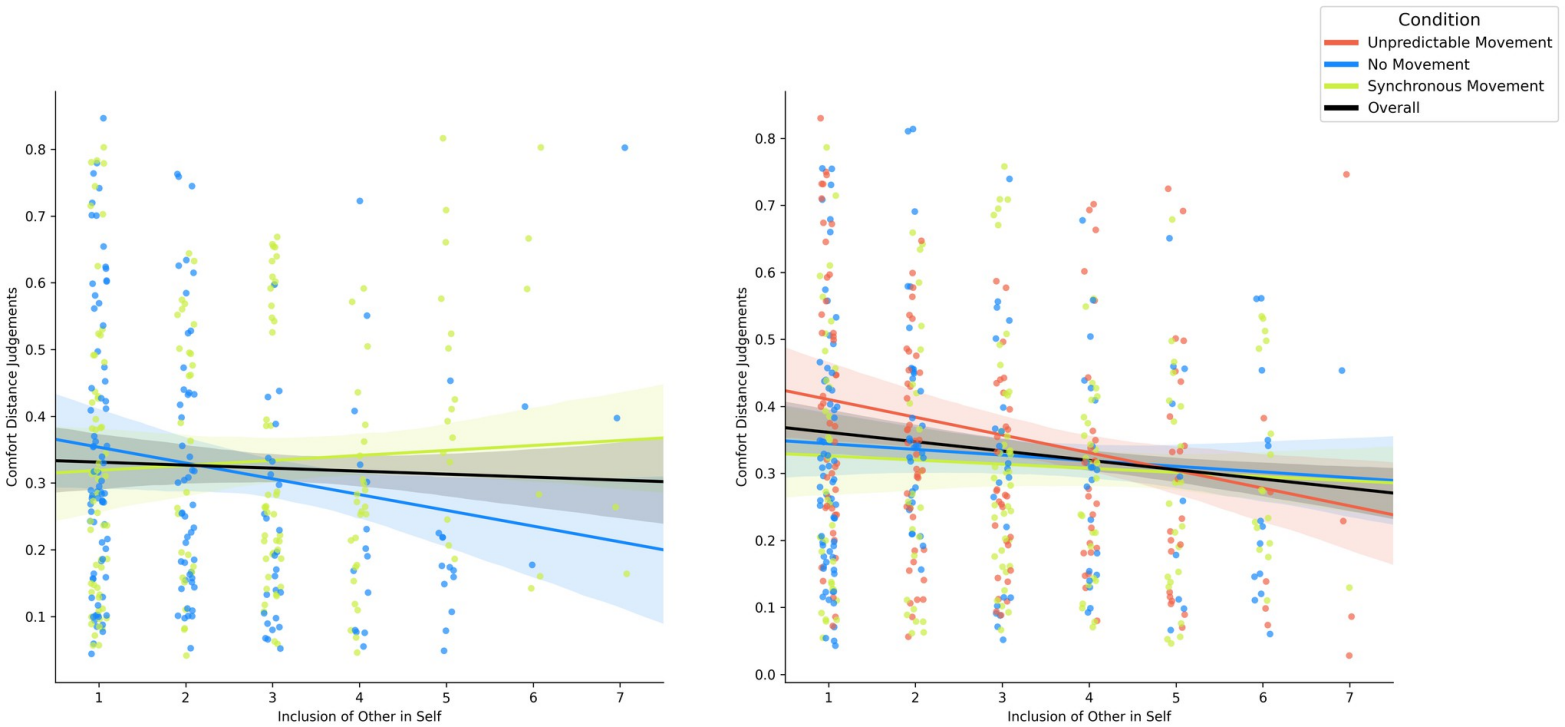
Correlation plots for Experiment 1 (left) and Experiment 2 (right) between self-other blurring (IOS scores) and comfort distance judgements.

To establish whether there was a relationship between self-other blurring and comfort distance judgments, we correlated IOS and CD Scores across participants. Since the data was not normally distributed, we report Spearman’s rank order correlation. Deviating from our previous findings, we found a significant correlation between IOS and mean comfort distance judgements [r = -0.117, 95% CI [-0.201, -0.030], p = 0.008], see Fig 5.

#### Post-task variables: Embodiment, strategy use and purpose check

We pre-registered the intention to compare if any of the post-task questions might explain some of the variance in the data. Specifically, we were interested in Embodiment, Strategy Use and Purpose Check. None of these factors had any significant impact on our variables of interest, for a detailed report please consult section 2 of the supplementary materials.

#### Pooling data from Experiment 1

In accordance with our pre-registration, we pooled the data from Synchronous movement and No Movement conditions with our data on these conditions from Experiment 1 to explore whether having a more powered study could elicit the effects. After pooling (N = 700), in accordance with the previous findings of our study while the IOS scores differed significantly across conditions, mean comfort judgements did not. IOS scores were not normally distributed according to the Shapiro-Wilk test (Sync W = 0.905, p < .001; No Movement W = 0.820, p < .001). Results showed that IOS scores were significantly higher in the Synchronous Movement condition (Sync: M = 2.90, Mdn = 3, SD = 1.59) as compared (U = 74,484, p < .001, Z = 4.95) to the No Movement condition (No Movement: M = 2.33, Mdn = 2, SD = 1.52) in a directional Mann-Whitney U test (Synchronous Movement > No Movement).

Mean Comfort Distance (CD) scores were not normally distributed according to the Shapiro-Wilk test (Sync W = 0.947, p < .001; NoMove W = 0.940, p < .001). Results showed that Mean Comfort Distances were not significantly lower in the Synchronous Movement condition (Sync: M = 0.32, Mdn = 0.28, SD = 0.19) as compared (U = 63074.0, p = 0.752, Z = 0.68) to the No Movement condition (No Movement: M = 0.31, Mdn = 0.28, SD = 0.19) in a directional Mann-Whitney U test (Synchronous Movement < No Movement).

Since the data was not normally distributed, we report Spearman’s rank order correlation. In the pooled data we found no significant correlation across participants between IOS and mean comfort distances [r = -0.051, 95% CI [0.024, -0.124], p = 0.181]. While the negative correlation is in the hypothesised direction, higher IOS reports are related to lower comfort distances, indicating the greater self-other blurring was related to allowing the group of avatars to approach at closer distances. However, this effect did not reach significance.

## Discussion

### Self-other blurring in mediated virtual synchrony

We hypothesised that synchronous jumping jacks movement between a desktop computer mediated avatar controlled by the participant and a group of virtual agents would elicit self-other blurring. Synchrony related self-other blurring has only been found either in real life settings wherein both participant and partner(s) are present [46, 58, 59] or the participant synchronises their own body movements to a partner presented on a computer screen or in VR setting [7, 18, 40, 41, 60, 61]. These paradigms involve the participant synchronising their own body movements, in situ, to a partner’s body movements. Thus, *it was yet to be established whether it is possible* to induce synchrony related affiliative tendencies in an experimental paradigm wherein the participant’s movement is translated onto an embodied avatar presented via a desktop computer. We found that synchronous movement between an avatar and a group of virtual agents leads to self-other blurring in online settings. In our first experiment, self-other blurring was greater in a shared synchronous movement condition as compared to a no movement condition. To the best of our knowledge, this is the first demonstration of self-other blurring through avatar controlled synchrony. It demonstrates that self-other blurring can occur in online settings through avatar-controlled synchrony, thereby extending the concept beyond real correspondence of movements. We replicated our finding in the second experiment where added an unpredictable movement condition to control for the possibility that presence of movement was the main driver of self-other blurring. Additionally, we found that synchronous movement led to greater self-other blurring in the shared synchronous movement condition as opposed to the unpredictable movement condition, indicating that predictable synchronous movement is associated with increased self-other blurring.

Online social psychological experiments overcome traditional challenges in the field by addressing issues such as limited sample sizes and lack of control [23]. In our experiment, we obtained an exceptionally large sample size (combined n = 870). As more individuals incorporate online environments into their daily lives, it becomes crucial to understand how our social and psychological cognitive processes translate to these settings. We induced movement synchrony between the participant’s avatar and an agent group. Depending on the experimental condition, an agent group facing the participants would either move in synchrony, remain still, or move unpredictably. Our discovery that a 3D desktop-computer mediated experience of synchrony leads to self-other blurring holds significant psychological implications. Firstly, it demonstrates that even in virtual environments, temporary groups can emerge as individuals readily adopt social cues of similarity, which are commonly employed in real-life interactions to identify common ground and in-group membership. Secondly, it indicates that movement similarity serves as a behavioural cue for in-group affiliation, even when the movement is not physically performed by the individual. One possible explanation for this indirect impact of synchrony is that participants project themselves onto a controlled avatar [62] and the correspondence of movement between this embodied avatar and a group of virtual agents can elicit self-other blurring, a psychological phenomenon linked to in-group categorisation. However, it is also plausible that simply tapping in synchrony with a group is sufficient to create self-other blurring, and the presence of the avatar may not be crucial. Additional research is required to explore this aspect further and gain a better understanding of the implications.

### Predictability of movement

The direct comparison of synchronous movement and unpredictable movement is novel. In their review of synchrony, Chartland & Lakin [57] define synchronous movement as comprising two major components: (1) movement identity and (2) temporal phasic (in-phase or anti-phase) relationship. Most synchrony paradigms focus on comparing and manipulating the latter of these components, that is, they create an asynchronous condition via an interacting group performing the same action at a different time/rhythm [32]. By introducing an unpredictable movement condition, we were able to tease out the contributions of the first component to synchrony—predictability of movement identity. Previous studies have noted that predictable movement identity plays a crucial role in interactional synchrony [63–65]. Our finding further consolidates this view by demonstrating that synchronous movement is associated with greater self-other blurring than unpredictable movement. Thus, predictable movement identity plays an important role in the affiliative tendencies that arise out of synchronous movement. However, the relationship between synchrony and predictability might be more multifaceted. A recent study by Ravery et al. found that both increasing complexity and synchrony were crucial for social bonding [66]. To further build on the specific contributions of movement identity, future experiments should focus on independently manipulating movement identity, complexity and timing.

### Interpersonal distance in online settings

Our paradigm did not elicit a reliable synchrony related differences in comfort distance judgements and we found inconsistent results regarding the relationship between our explicit measure of self-other blurring and implicit measure of comfort distance judgements. Specifically, the measures showed a significant correlation in our second experiment but not in the first. Further, in Experiment 1 we found a significant correlation when only data from the no movement condition were analysed.

We obtained mixed results regarding the association between explicit self-other blurring and implicit comfort distance judgments. In our initial experiment, we found no significant correlation between self-other blurring and comfort distance judgments across participants. However, when we examined the data by condition, we discovered a significant negative correlation in the no movement condition. This aligns with our hypothesis that agents inducing a higher degree of self-other blurring would be allowed to approach closer to the participants’ avatars. Experiment 2 provided further support for this finding when we analysed the data across conditions. This suggests that there may be a connection between self-other blurring and implicit comfort distance judgments, but this relationship is not consistently observed across different conditions. There are two possible ways to interpret this finding, (1) indirect desktop mediated synchrony does not elicit implicit effects of proxemics or (2) our measure of proxemics does not sufficiently capture this effect. Given that naturalistic movement synchrony experiments have successfully demonstrated the impact of synchrony on preferred proximity [5, 21], it is plausible that there exists a general limitation whereby the use of virtual environments and avatars cannot replicate the proxemics-related differences associated with synchrony. In line with this view, another study utilising virtual reality (VR) as part of their research, which included physical proximity as a dependent measure (calculated as the average distance maintained throughout the experiment), found no significant differences in physical proximity [61]. Thus, there may be aspects of affiliative tendencies that arise from synchrony, which cannot be effectively replicated in online or VR settings. Although the current evidence points in this direction, further empirical studies are needed to firmly establish that proxemic differences cannot be replicated in VR or other mediated virtual experiences.

### Implications for virtual synchrony and future directions

It is possible that the influence of synchrony on proxemics is difficult to capture in an online setting, and it might be more reliable to elicit this effect in a virtual reality (VR) environment. Given that the significant correlations align with our initial hypothesis, conducting another study in VR with varied experimental settings could provide a more robust test of this hypothesis. There are significant distinctions between online 3D desktop environments and VR settings. Cohen et al. discovered that when participants listened to a person sharing a painful autobiographical memory in VR, as opposed to on a computer, there was a greater sense of social presence, affiliative feelings, and facial synchrony in the VR setup [67]. Recreating our experiment in VR could potentially amplify the effects of synchronous movement, as participants would need to engage in real movement correspondence with an agent group. Furthermore, employing a VR environment for the comfort distance under the passive approach task is likely to enhance immersion, thereby increasing the potential for more pronounced implicit effects on comfort distance judgments.

In conclusion, we found that avatar-mediated synchrony with a group of virtual agents leads to self-other blurring, suggesting that real movement correspondence with a synchrony partner is not a prerequisite for in-group bond formation. We also found that predictability of movement identity contributes to self-other blurring by directly comparing synchronous movement to an unpredictable movement condition. By demonstrating that synchronised movement’s psychological effects can be recreated in virtual environments, this research highlights the potential for fostering group cohesion in online team-building and collaboration. Our findings suggest that even without physical presence, virtual spaces can support the development of cohesive online communities.

## Supporting information

S1 File This document contains detailed analyses and data regarding Experiment 1 and Experiment 2 post-task variables, including embodiment, strategy use, and purpose check. It provides statistical comparisons of dependent variables in relation to attention checks, strategy use, and purpose.
(DOCX)

## References

[1] Van Ulzen NR, Lamoth CJC, Daffertshofer A, Semin GR, Beek PJ. Characteristics of instructed and uninstructed interpersonal coordination while walking side-by-side. Neurosci Lett. 2008;432: 88–93. doi: 10.1016/j.neulet.2007.11.070 18242846

[2] Páez D, Rimé B, Basabe N, Wlodarczyk A, Zumeta L. Psychosocial effects of perceived emotional synchrony in collective gatherings. J Pers Soc Psychol. 2015;108: 711–729. doi: 10.1037/pspi0000014 25822033

[3] Durkheim E. The elementary forms of the religious life. 1912.

[4] Dunbar RIM. Coevolution of neocortical size, group size and language in humans. Behav Brain Sci. 1993;16: 681–694. doi: 10.1017/S0140525X00032325

[5] Jackson JC, Jong J, Bilkey D, Whitehouse H, Zollmann S, McNaughton C, et al. Synchrony and Physiological Arousal Increase Cohesion and Cooperation in Large Naturalistic Groups. Sci Rep. 2018;8: 127. doi: 10.1038/s41598-017-18023-4 29317675 PMC5760525

[6] Lakens D. Movement synchrony and perceived entitativity. J Exp Soc Psychol. 2010;46: 701–708. doi: 10.1016/j.jesp.2010.03.015

[7] Launay J, Dean RT, Bailes F. Synchronising movements with the sounds of a virtual partner enhances partner likeability. Cogn Process. 2014;15: 491–501. doi: 10.1007/s10339-014-0618-0 24805849

[8] Loersch C, Arbuckle NL. Unraveling the mystery of music: Music as an evolved group process. J Pers Soc Psychol. 2013;105: 777–798. doi: 10.1037/a0033691 23895270

[9] Pearce E, Launay J, Dunbar RIM. The ice-breaker effect: singing mediates fast social bonding. R Soc Open Sci. 2015;2: 150221. doi: 10.1098/rsos.150221 26587241 PMC4632513

[10] Shultz S, Dunbar R. Encephalization is not a universal macroevolutionary phenomenon in mammals but is associated with sociality. Proc Natl Acad Sci. 2010;107: 21582–21586. doi: 10.1073/pnas.1005246107 21098277 PMC3003036

[11] Vuoskoski JK, Clarke EF, DeNora T. Music listening evokes implicit affiliation. Psychol Music. 2017;45: 584–599. doi: 10.1177/0305735616680289

[12] Weinstein D, Launay J, Pearce E, Dunbar RIM, Stewart L. Singing and social bonding: changes in connectivity and pain threshold as a function of group size. Evol Hum Behav. 2016;37: 152–158. 27158219 10.1016/j.evolhumbehav.2015.10.002 PMC4856205

[13] Cross L, Turgeon M, Atherton G. How Moving Together Binds Us Together: The Social Consequences of Interpersonal Entrainment and Group Processes. Open Psychol. 2019;1: 273–302. doi: 10.1515/psych-2018-0018

[14] Tunçgenç B, Cohen E. Interpersonal movement synchrony facilitates pro-social behavior in children’s peer-play. Dev Sci. 2018;21: e12505. doi: 10.1111/desc.12505 27990719

[15] Cirelli LK. How interpersonal synchrony facilitates early prosocial behavior. Curr Opin Psychol. 2018;20: 35–39. doi: 10.1016/j.copsyc.2017.08.009 28830004

[16] Atherton G, Cross L. Walking in My Shoes: Imagined Synchrony Improves Attitudes Towards Out-groups. Psychol Stud. 2020;65: 351–359. doi: 10.1007/s12646-020-00568-6

[17] Cross L, Whiteman L, Ward S, Atherton G. Moving From Me to We: Interpersonal Coordination’s Effects on Self-Construal. Open Psychol. 2021;3: 50–63. doi: 10.1515/psych-2020-0110

[18] Miles LK, Lumsden J, Richardson MJ, Neil Macrae C. Do birds of a feather move together? Group membership and behavioral synchrony. Exp Brain Res. 2011;211: 495–503. doi: 10.1007/s00221-011-2641-z 21448575

[19] Good A, Choma B, Russo FA. Movement Synchrony Influences Intergroup Relations in a Minimal Groups Paradigm. Basic Appl Soc Psychol. 2017;39: 231–238. doi: 10.1080/01973533.2017.1337015

[20] Reddish P, Tong EMW, Jong J, Lanman JA, Whitehouse H. Collective synchrony increases prosociality towards non-performers and outgroup members. Br J Soc Psychol. 2016;55: 722–738. doi: 10.1111/bjso.12165 27683102

[21] Tunçgenç B, Cohen E. Movement Synchrony Forges Social Bonds across Group Divides. Front Psychol. 2016;7. doi: 10.3389/fpsyg.2016.00782 27303341 PMC4882973

[22] Teneggi C, Canzoneri E, di Pellegrino G, Serino A. Social Modulation of Peripersonal Space Boundaries. Curr Biol. 2013;23: 406–411. doi: 10.1016/j.cub.2013.01.043 23394831

[23] Blascovich J, Loomis J, Beall AC, Swinth KR, Hoyt CL, Bailenson JN. TARGET ARTICLE: Immersive Virtual Environment Technology as a Methodological Tool for Social Psychology. Psychol Inq. 2002;13: 103–124. doi: 10.1207/S15327965PLI1302_01

[24] Iachini T, Coello Y, Frassinetti F, Ruggiero G. Body space in social interactions: A comparison of reaching and comfort distance in immersive virtual reality. PLoS ONE. 2014;9. doi: 10.1371/journal.pone.0111511 25405344 PMC4236010

[25] Naito A, Go K, Shima H, Kijima A. Synchrony in triadic jumping performance under the constraints of virtual reality. Sci Rep. 2022;12: 12417. doi: 10.1038/s41598-022-16703-4 35859003 PMC9297677

[26] Tarr B, Slater M, Cohen E. Synchrony and social connection in immersive Virtual Reality. Sci Rep. 2018;8: 3693. doi: 10.1038/s41598-018-21765-4 29487405 PMC5829252

[27] Dotsch R, Wigboldus DHJ. Virtual prejudice. J Exp Soc Psychol. 2008;44: 1194–1198. doi: 10.1016/j.jesp.2008.03.003

[28] McCall C. Mapping Social Interactions: The Science of Proxemics. In: Wöhr M, Krach S, editors. Social Behavior from Rodents to Humans. Cham: Springer International Publishing; 2017. pp. 295–308. 10.1007/7854_2015_431 26728171

[29] Fini C, Brass M, Committeri G. Social scaling of extrapersonal space: Target objects are judged as closer when the reference frame is a human agent with available movement potentialities. Cognition. 2015;134: 50–56. doi: 10.1016/j.cognition.2014.08.014 25460378

[30] Maister L, Cardini F, Zamariola G, Serino A, Tsakiris M. Your place or mine: Shared sensory experiences elicit a remapping of peripersonal space. Neuropsychologia. 2015;70: 455–461. doi: 10.1016/j.neuropsychologia.2014.10.027 25447370

[31] Aron A, Aron EN, Smollan D. Inclusion of Other in the Self Scale and the structure of interpersonal closeness. J Pers Soc Psychol. 1992;63: 596–612. doi: 10.1037/0022-3514.63.4.596

[32] Reddish P, Bulbulia J, Fischer R. Does synchrony promote generalized prosociality? Relig Brain Behav. 2014;4: 3–19. doi: 10.1080/2153599X.2013.764545

[33] Tarr B, Launay J, Cohen E, Dunbar R. Synchrony and exertion during dance independently raise pain threshold and encourage social bonding. Biol Lett. 2015;11: 20150767. doi: 10.1098/rsbl.2015.0767 26510676 PMC4650190

[34] Bakan D. Duality of human existence: an essay on psychology and religion. Boston: Beacon Press; 1966.

[35] Good A, Russo FA. Singing Promotes Cooperation in a Diverse Group of Children. Soc Psychol. 2016;47: 340–344. doi: 10.1027/1864-9335/a000282

[36] Hove MJ, Risen JL. It’s All in the Timing: Interpersonal Synchrony Increases Affiliation. Soc Cogn. 2009;27: 949–960. doi: 10.1521/soco.2009.27.6.949

[37] Rabinowitch T-C, Knafo-Noam A. Synchronous Rhythmic Interaction Enhances Children’s Perceived Similarity and Closeness towards Each Other. Kotz S, editor. PLOS ONE. 2015;10: e0120878. doi: 10.1371/journal.pone.0120878 25853859 PMC4390221

[38] Valdesolo P, DeSteno D. Synchrony and the social tuning of compassion. Emotion. 2011;11: 262–266. doi: 10.1037/a0021302 21500895

[39] Woolhouse MH, Lai R. Traces across the body: the influence of music-dance synchrony on the observation of dance. Front Hum Neurosci. 2014;8. doi: 10.3389/fnhum.2014.00965 25520641 PMC4253660

[40] Lumsden J, Miles LK, Richardson MJ, Smith CA, Macrae CN. Who syncs? Social motives and interpersonal coordination. J Exp Soc Psychol. 2012;48: 746–751. doi: 10.1016/j.jesp.2011.12.007

[41] Lumsden J, Miles LK, Macrae CN. Sync or sink? Interpersonal synchrony impacts self-esteem. Front Psychol. 2014;5. doi: 10.3389/fpsyg.2014.01064 25285090 PMC4168669

[42] Cirelli LK, Einarson KM, Trainor LJ. Interpersonal synchrony increases prosocial behavior in infants. Dev Sci. 2014;17: 1003–1011. doi: 10.1111/desc.12193 25513669

[43] Cirelli LK, Wan SJ, Trainor LJ. Social Effects of Movement Synchrony: Increased Infant Helpfulness only Transfers to Affiliates of Synchronously Moving Partners. Infancy. 2016;21: 807–821. doi: 10.1111/infa.12140

[44] Cirelli LK, Wan SJ, Spinelli C, Trainor LJ. Effects of Interpersonal Movement Synchrony on Infant Helping Behaviors. Music Percept. 2017;34: 319–326. doi: 10.1525/mp.2017.34.3.319

[45] Cirelli LK, Wan SJ, Trainor LJ. Fourteen-month-old infants use interpersonal synchrony as a cue to direct helpfulness. Philos Trans R Soc B Biol Sci. 2014;369: 20130400. doi: 10.1098/rstb.2013.0400 25385778 PMC4240967

[46] Tarr B, Launay J, Dunbar RIM. Silent disco: dancing in synchrony leads to elevated pain thresholds and social closeness. Evol Hum Behav. 2016;37: 343–349. doi: 10.1016/j.evolhumbehav.2016.02.004 27540276 PMC4985033

[47] Anshel A, Kipper DA. The Influence of Group Singing on Trust and Cooperation. J Music Ther. 1988;25: 145–155. doi: 10.1093/jmt/25.3.145

[48] Pearce E, Launay J, Van Duijn M, Rotkirch A, David-Barrett T, Dunbar RIM. Singing together or apart: The effect of competitive and cooperative singing on social bonding within and between sub-groups of a university Fraternity. Psychol Music. 2016;44: 1255–1273. doi: 10.1177/0305735616636208 27777494 PMC5074360

[49] Rabinowitch T-C, Meltzoff AN. Joint Rhythmic Movement Increases 4-Year-Old Children’s Prosocial Sharing and Fairness Toward Peers. Front Psychol. 2017;8: 1050. doi: 10.3389/fpsyg.2017.01050 28694786 PMC5483466

[50] Cross L, Michael J, Wilsdon L, Henson A, Atherton G. Still want to help? Interpersonal coordination’s effects on helping behaviour after a 24 hour delay. Acta Psychol (Amst). 2020;206: 103062. doi: 10.1016/j.actpsy.2020.103062 32442775

[51] Fujiwara K, Hoegen R, Gratch J, Dunbar NE. Synchrony facilitates altruistic decision making for non-human avatars. Comput Hum Behav. 2022;128: 107079. doi: 10.1016/j.chb.2021.107079

[52] Lang M, Bahna V, Shaver JH, Reddish P, Xygalatas D. Sync to link: Endorphin-mediated synchrony effects on cooperation. Biol Psychol. 2017;127: 191–197. doi: 10.1016/j.biopsycho.2017.06.001 28596129

[53] Fessler DMT, Holbrook C. Marching into battle: synchronized walking diminishes the conceptualized formidability of an antagonist in men. Biol Lett. 2014;10: 20140592. doi: 10.1098/rsbl.2014.0592 25165456 PMC4155918

[54] Reddish P, Fischer R, Bulbulia J. Let’s Dance Together: Synchrony, Shared Intentionality and Cooperation. PLOS ONE. 2013;8: e71182. doi: 10.1371/journal.pone.0071182 23951106 PMC3737148

[55] Cross L, Atherton G, Sebanz N. Intentional synchronisation affects automatic imitation and source memory. Sci Rep. 2021;11: 573. doi: 10.1038/s41598-020-79796-9 33436752 PMC7804244

[56] Iachini T, Coello Y, Frassinetti F, Ruggiero G. Body Space in Social Interactions: A Comparison of Reaching and Comfort Distance in Immersive Virtual Reality. Kotz S, editor. PLoS ONE. 2014;9: e111511. doi: 10.1371/journal.pone.0111511 25405344 PMC4236010

[57] Chartrand TL, Lakin JL. The Antecedents and Consequences of Human Behavioral Mimicry. Annu Rev Psychol. 2013;64: 285–308. doi: 10.1146/annurev-psych-113011-143754 23020640

[58] Paxton A, Dale R. Argument disrupts interpersonal synchrony. Q J Exp Psychol. 2013;66: 2092–2102. doi: 10.1080/17470218.2013.853089 24303888

[59] Valdesolo P, Ouyang J, DeSteno D. The rhythm of joint action: Synchrony promotes cooperative ability. J Exp Soc Psychol. 2010;46: 693–695. doi: 10.1016/j.jesp.2010.03.004

[60] Launay J, Dean RT, Bailes F. Synchronization can influence trust following virtual interaction. Exp Psychol. 2013;60: 53–63. doi: 10.1027/1618-3169/a000173 22935329

[61] Tarr B, Slater M, Cohen E. Synchrony and social connection in immersive Virtual Reality. Sci Rep. 2018;8: 3693. doi: 10.1038/s41598-018-21765-4 29487405 PMC5829252

[62] Fribourg R, Argelaguet F, Lecuyer A, Hoyet L. Avatar and Sense of Embodiment: Studying the Relative Preference Between Appearance, Control and Point of View. IEEE Trans Vis Comput Graph. 2020;26: 2062–2072. doi: 10.1109/TVCG.2020.2973077 32070975

[63] Knoblich G, Sebanz N. The Social Nature of Perception and Action. Curr Dir Psychol Sci. 2006;15: 99–104. doi: 10.1111/j.0963-7214.2006.00415.x

[64] Marsh KL, Richardson MJ, Schmidt RC. Social Connection Through Joint Action and Interpersonal Coordination. Top Cogn Sci. 2009;1: 320–339. doi: 10.1111/j.1756-8765.2009.01022.x 25164936

[65] Sebanz N, Knoblich G. Prediction in Joint Action: What, When, and Where. Top Cogn Sci. 2009;1: 353–367. doi: 10.1111/j.1756-8765.2009.01024.x 25164938

[66] Ravreby I, Shilat Y, Yeshurun Y. Liking as a balance between synchronization, complexity and novelty. Sci Rep. 2022;12: 3181. doi: 10.1038/s41598-022-06610-z 35210459 PMC8873358

[67] Cohen D, Landau DH, Friedman D, Hasler BS, Levit-Binnun N, Golland Y. Exposure to social suffering in virtual reality boosts compassion and facial synchrony. Comput Hum Behav. 2021;122: 106781. doi: 10.1016/j.chb.2021.106781

