# Supplementary material for: Syncing online: A methodological investigation into movement synchrony, proxemics, and self-other blurring in virtual spaces
Source: PLoS One. 2024 Oct 22;19(10):e0308843. doi: 10.1371/journal.pone.0308843 (PMC11495584; doi:10.1371/journal.pone.0308843)
Supplement: S1 File — It provides statistical comparisons of dependent variables in relation to attention checks, strategy use, and purpose. (DOCX) [file pone.0308843.s001.docx]

**Supplementary Materials: Syncing Online: A Methodological Investigation into Movement Synchrony, Proxemics, and Self-Other Blurring in Virtual Spaces**

We assessed whether any of the post-task questions could elucidate a portion of the variability in the data. More specifically, our interest lay in exploring Embodiment, Strategy Use, and Purpose Check.

**Section 1:**

**Experiment 1 Post-Task Variables: Embodiment, Strategy Use and Purpose Check**

To check whether participants were able to correctly identify their avatar during the experiment as a form of attention check, we asked them to report the shirt colour of their avatar. This was always white and the agent group was always wearing blue. While most participants were accurate (53.3%), a substantial number of participants reported the colour of the agent group (43.3%). We conducted an independent samples non-parametric t-test to check whether there was a difference in our dependent variables in correspondence with these choices. Comfort distance: Attention-check passed [M = 0.334, SD = 0.202, SE = 0.0162] as compared to the Attention check not passed [M= 0.310, SD = 0.196, SE= 0.0139] [t(330) = 1.11,p = 0.265, d= 0.119, 95% CI -0.0180, 0.0656]. IOS: Attention check passed [M = 2.35, SD =1.57, SE =0.125] as compared to the Attention check not passed [M= 2.46, SD = 1.40, SE= 0.099] [t_(315)_ = -0.719,p = 0.473, d= -0.0772, 95% CI -0.429, 0.199]. Thus, passing the attention check by choosing blue for the embodiment check question did not have any significant impact in our variables of interest.

We also asked participants to report whether they used a strategy or were able to figure out the purpose of the experiment, 69.7% reported that they did not. There was no significant difference caused by reported strategy use, Comfort distance: Strategy Used [M = 0.298, SD = 0.191, SE = 0.0183] as compared to the No Strategy Used [M= 0.328, SD = 0.282, SE= 0.0126][t_(214)_ = 1.35,p = 0.178, d= 0.153, 95% CI -0.0138, 0.0740]. IOS: Strategy Used [M = 2.46, SD =1.65, SE =0.158] as compared to the No Strategy Used [M= 2.39, SD = 1.39, SE= 0.087] [t_(177)_ = -0.378,p = 0.706, d= -0.0448, 95% CI -0.425, 0.289]. 83.3% of participants reported that they did not know the purpose of the experiment. There was no significant difference caused by reported purpose understanding. Upon further inspection of the responses provided as to the purpose of the experiment by the remaining 16.7%, none of the participants were able to accurately report the purpose of the experiment.

**Section 2:**

**Experiment 2 Post-Task Variables: Embodiment, Strategy Use and Purpose Check**

To check whether participants were able to correctly identify their avatar during the experiment as a form of attention check, we asked them to report the shirt colour of their avatar. This was always white and the agent group was always wearing blue. While most participants were accurate (52.7%), a substantial number of participants reported the colour of the agent group (35.1%). Relatively few participants answered Red (2.5%), Yellow (2.9%) and Green (6.7%), We conducted an independent samples non-parametric t-test to check whether there was a difference in our dependent variables in correspondence with these choices. Comfort distance: Attention check passed [M = 0.334, SD = 0.202, SE = 0.0162] as compared to the Attention check not passed [M= 0.310, SD = 0.196, SE= 0.0139] [t_(330)_ = 1.11,p = 0.265, d= 0.119, 95% CI -0.0180, 0.0656]. IOS: Attention check passed [M = 2.35, SD =1.57, SE =0.125] as compared to the Attention check not passed [M= 2.46, SD = 1.40, SE= 0.099] [t_(315)_ = -0.719,p = 0.473, d= -0.0772, 95% CI -0.429, 0.199].

We asked participants to report whether they used a strategy during the experiment, 78.2% reported that they did not. There was no significant difference caused by reported strategy use, Comfort distance: Strategy Used [M = 0.320, SD = 0.175, SE = 0.0168] as compared to the No Strategy Used [M= 0.323, SD = 0.182, SE= 0.009] [t_(177)_ = -0.143,p = 0.889 d= -0.154, 95% CI -0.04, 0.0351]. IOS: Strategy Used [M = 2.75, SD =1.69, SE =0.162] as compared to the No Strategy Used [M= 2.85, SD = 1.63, SE= 0.083] [t_(167)_ = -0.536,p = 0.593, d= -0.0589, 95% CI -0.457 0.262].

85% of participants reported that they did not know the purpose of the experiment. There was no significant difference caused by reported purpose understanding. Upon further inspection of the responses provided as to the purpose of the experiment by the remaining 15%, none of the participants were able to accurately discover the purpose of the experiment.
